# Predictors of mortality in subjects with progressive fibrosing interstitial lung diseases

**DOI:** 10.1111/resp.14231

**Published:** 2022-02-27

**Authors:** Kevin K. Brown, Yoshikazu Inoue, Kevin R. Flaherty, Fernando J. Martinez, Vincent Cottin, Francesco Bonella, Stefania Cerri, Sonye K. Danoff, Stephane Jouneau, Rainer‐Georg Goeldner, Martin Schmidt, Susanne Stowasser, Rozsa Schlenker‐Herceg, Athol U. Wells

**Affiliations:** ^1^ Department of Medicine National Jewish Health Denver Colorado USA; ^2^ Clinical Research Center National Hospital Organization Kinki‐Chuo Chest Medical Center Sakai City Japan; ^3^ Division of Pulmonary and Critical Care Medicine University of Michigan Ann Arbor Michigan USA; ^4^ Weill Cornell Medicine New York New York USA; ^5^ National Reference Centre for Rare Pulmonary Diseases, Louis Pradel Hospital, Hospices Civils de Lyon Claude Bernard University Lyon 1 Lyon France; ^6^ Center for Interstitial and Rare Lung Diseases, Department of Pneumology, Ruhrlandklinik University Hospital University of Duisburg‐Essen Essen Germany; ^7^ Center for Rare Lung Disease Azienda Ospedaliero‐Universitaria Policlinico di Modena Modena Italy; ^8^ Johns Hopkins Medicine Baltimore Maryland USA; ^9^ Department of Respiratory Medicine Competences Centre for Rare Pulmonary Diseases, CHU Rennes, IRSET UMR 1085, Univ Rennes Rennes France; ^10^ Boehringer Ingelheim Pharma GmbH & Co. KG Biberach Germany; ^11^ Boehringer Ingelheim Pharma GmbH Ingelheim am Rhein Germany; ^12^ Boehringer Ingelheim International GmbH Ingelheim am Rhein Germany; ^13^ Boehringer Ingelheim Pharmaceuticals, Inc. Ridgefield Connecticut USA; ^14^ National Institute for Health Research Respiratory Biomedical Research Unit, Royal Brompton and Harefield NHS Foundation Trust, and National Heart and Lung Institute Imperial College London UK

**Keywords:** clinical trial, death, fibrosing interstitial lung disease, forced vital capacity, pulmonary fibrosis, pulmonary function test

## Abstract

**Background and objective:**

Demographic and clinical variables, measured at baseline or over time, have been associated with mortality in subjects with progressive fibrosing interstitial lung diseases (ILDs). We used data from the INPULSIS trials in subjects with idiopathic pulmonary fibrosis (IPF) and the INBUILD trial in subjects with other progressive fibrosing ILDs to assess relationships between demographic/clinical variables and mortality.

**Methods:**

The relationships between baseline variables and time‐varying covariates and time to death over 52 weeks were analysed using pooled data from the INPULSIS trials and, separately, the INBUILD trial using a Cox proportional hazards model.

**Results:**

Over 52 weeks, 68/1061 (6.4%) and 33/663 (5.0%) subjects died in the INPULSIS and INBUILD trials, respectively. In the INPULSIS trials, a relative decline in forced vital capacity (FVC) >10% predicted within 12 months (hazard ratio [HR] 3.77) and age (HR 1.03 per 1‐year increase) were associated with increased risk of mortality, while baseline FVC % predicted (HR 0.97 per 1‐unit increase) and diffusing capacity of the lungs for carbon monoxide (DLCO) % predicted (HR 0.77 per 1‐unit increase) were associated with lower risk. In the INBUILD trial, a relative decline in FVC >10% predicted within 12 months (HR 2.60) and a usual interstitial pneumonia‐like fibrotic pattern on HRCT (HR 2.98) were associated with increased risk of mortality, while baseline DLCO % predicted (HR 0.95 per 1‐unit increase) was associated with lower risk.

**Conclusion:**

These data support similarity in the course of lung injury between IPF and other progressive fibrosing ILDs and the value of FVC decline as a predictor of mortality.

## Abstract 2

We assessed relationships between baseline and time‐varying factors and mortality over 52 weeks in 1061 subjects with idiopathic pulmonary fibrosis (IPF) and 663 subjects with other progressive fibrosing interstitial lung diseases (ILDs). Our findings support similarity in the course of IPF and ILD and an association between decline in forced vital capacity and mortality.

## INTRODUCTION

Idiopathic pulmonary fibrosis (IPF) is, by definition, a progressive fibrosing interstitial lung disease (ILD).^1^ Other chronic fibrosing ILDs may also develop a progressive phenotype characterized by an increasing extent of fibrotic features on HRCT; declining lung function; worsening symptoms, functional impairment and quality of life; and early mortality.^2^, ^3^, ^4^

In the INPULSIS trials in subjects with IPF, and the INBUILD trial in subjects with fibrosing ILDs other than IPF who had shown progression within the previous 2 years despite management deemed appropriate in clinical practice, nintedanib reduced the rate of decline in forced vital capacity (FVC) (ml/year) over 52 weeks by approximately 50% compared to placebo.^5^, ^6^, ^7^ In the placebo groups of these trials, the rate of decline in FVC and the risk of mortality were similar and a relative decline in FVC of >10% of the predicted value was associated with a more than three‐fold increase in the risk of death over 52 weeks.^8^

In addition to a decline in FVC, in subjects with fibrosing ILDs, other demographic/clinical characteristics have been associated with mortality, including higher age, more severely impaired lung function and the presence of honeycombing or traction bronchiectasis on HRCT.^3^, ^9^, ^10^, ^11^, ^12^, ^13^, ^14^, ^15^, ^16^, ^17^, ^18^, ^19^, ^20^ We used data from the INPULSIS and INBUILD trials to assess the relationship between demographic/clinical variables and mortality and to determine the relative importance of a decline in FVC after accounting for these variables.

## METHODS

### INBUILD and INPULSIS trials

The designs of the INBUILD trial (NCT02999178) and INPULSIS trials (NCT01335464, NCT01335477) have been published and the protocols are publicly available.^5^, ^6^ In brief, subjects in the INPULSIS trials were aged ≥40 years, with IPF, FVC ≥50% predicted and diffusing capacity of the lungs for carbon monoxide (DLCO) ≥30%–<80% predicted.^5^ Subjects in the INBUILD trial were aged ≥18 years; had a chronic fibrosing ILD other than IPF; met criteria for ILD progression within the previous 2 years, despite management deemed appropriate in clinical practice; had FVC ≥45% predicted; and DLCO ≥30%–<80% predicted.^6^ The INBUILD and INPULSIS trials were conducted in accordance with the principles of the Declaration of Helsinki and the Harmonized Tripartite Guideline for Good Clinical Practice from the International Conference on Harmonization, and were approved by local authorities.

### Data analysis

We analysed the relationship between baseline variables and time‐varying covariates (Table 1) and time to death over 52 weeks in the INBUILD trial and, separately, in pooled data from the INPULSIS trials. Subjects who received ≥1 dose of nintedanib or placebo were included. Associations between variables and mortality were assessed using a Cox proportional hazards model where time‐varying covariates were included using the programming statements method; missing data were not imputed.

**TABLE 1:** Overview of variables

Description	Nature
HRCT pattern (UIP‐like fibrotic pattern; other fibrotic patterns)^a^	Baseline
Relative decline in DLCO^b^>15% predicted within 12 months	Time‐varying
Relative decline in FVC >10% predicted with 12 months	Time‐varying
Relative decline in weight >5% within 12 months	Time‐varying
Age (years)	Baseline
Sex	Baseline
Treatment (nintedanib; placebo)	Baseline
FVC, ml	Baseline
FVC % predicted	Baseline
DLCO % predicted^b^	Baseline
Race (Asian; Black/African American; White)	Baseline
BMI (kg/m^2^)	Baseline
Tobacco consumption (current and former; never)	Baseline
Time since diagnosis (years)	Baseline
ILD diagnosis (iNSIP, unclassifiable IIP, HP, autoimmune ILDs, other ILDs)^a^	Baseline

^a^ INBUILD trial only.

^b^ Corrected for haemoglobin.

Abbreviations: DLCO, diffusing capacity of the lungs for carbon monoxide; FVC, forced vital capacity; HP, hypersensitivity pneumonitis; IIP, idiopathic interstitial pneumonia; ILD, interstitial lung disease; iNSIP, idiopathic non‐specific interstitial pneumonia; UIP, usual interstitial pneumonia.

The main model selection algorithm employed was ‘stepwise selection’, but ‘forward selection’ and ‘backward elimination’ were also utilized. Change in the Akaike information criterion was used to determine important variables. The threshold to include or remove a variable was *p* = 0.05. To evaluate the impact of a selected variable on mortality, hazard ratios (HRs) and their CIs were calculated. HRs for continuous variables reflect the impact of a unit change in the underlying variable on mortality. HRs for categorical variables reflect the impact on mortality of being in a defined group compared with the reference group, or, for time‐varying covariates, of having the defined decline compared with not having that decline. Sensitivity analyses based on multiple imputation, jump to reference and copy reference^21^ were conducted to determine whether the findings were robust irrespective of the amount of missing data or the imputation method used.

To identify the threshold of FVC decline that was associated with the highest risk of death, the relationship between three alternative measures of FVC decline (relative decline in FVC >10% predicted within 6 months and relative decline in FVC >5% predicted within 6 and 12 months) and mortality was evaluated by: (i) replacing decline in FVC >10% predicted within 12 months with one of the alternative FVC decline measures in a model that included all other variables; (ii) including all four decline in FVC covariates in a model with all other variables; and (iii) including all four decline in FVC covariates in a model without any other variables. The decline in FVC and mortality were assessed within the same time period (i.e., 12 months).

## RESULTS

### Study participants

In total, 1061 and 663 subjects received ≥1 dose of nintedanib or placebo in the INPULSIS and INBUILD trials, respectively. Their baseline characteristics have been described.^5^, ^6^

Over 52 weeks, 68 subjects (6.4%) died in the INPULSIS trials and 33 subjects (5.0%) died in the INBUILD trial (27 [6.6%] subjects with a usual interstitial pneumonia [UIP]‐like fibrotic pattern on HRCT and six [2.4%] subjects with other fibrotic patterns on HRCT).

### Selected variables and their association with mortality

In the INPULSIS trials, a relative decline in FVC >10% predicted within 12 months, FVC % predicted at baseline, age and DLCO % predicted at baseline were selected as variables associated with mortality (see Table S1 in the Supporting Information). A relative decline in FVC >10% predicted within 12 months (HR 3.77 [95% CI: 2.28, 6.24]) and older age (HR 1.03 [95% CI: 1.00, 1.07] per 1‐year increase) were associated with a significantly increased risk of mortality, whereas higher FVC % predicted at baseline (HR 0.97 [95% CI: 0.95, 0.99] per 1‐unit increase) and higher DLCO % predicted at baseline (HR 0.77 [95% CI: 0.61, 0.98] per 1‐unit increase) were associated with a lower risk of mortality (Table 2).

**TABLE 2:** Association between selected variables and mortality based on data from the INPULSIS and INBUILD trials

	HR (95% CI)	*p*‐value
Selected variables in INPULSIS		
Relative decline in FVC >10% predicted within 12 months	3.77 (2.28, 6.24)	<0.0001
Age	1.03 (1.00, 1.07)	0.037
FVC % predicted at baseline	0.97 (0.95, 0.99)	0.0007
DLCO % predicted at baseline	0.77 (0.61, 0.98)	0.033
Selected variables in INBUILD		
HRCT pattern^a^	2.98 (1.23, 7.22)	0.016
Relative decline in FVC >10% predicted within 12 months	2.60 (1.28, 5.31)	0.0085
DLCO % predicted at baseline	0.95 (0.91, 0.98)	0.0046

Abbreviations: DLCO, diffusing capacity of the lungs for carbon monoxide; FVC, forced vital capacity; HR, hazard ratio; UIP, usual interstitial pneumonia.

^a^ HR for subjects with a UIP‐like fibrotic pattern on HRCT compared with subjects with other fibrotic patterns on HRCT.

In the INBUILD trial, a relative decline in FVC >10% predicted within 12 months, DLCO % predicted at baseline and HRCT pattern were selected as variables associated with mortality (see Table S2 in the Supporting Information). A relative decline in FVC >10% predicted within 12 months (HR 2.60 [95% CI: 1.28, 5.31]) and a UIP‐like fibrotic pattern on HRCT versus other fibrotic patterns (HR 2.98 [95% CI: 1.23, 7.22]) were associated with an increased risk of mortality, while higher DLCO % predicted at baseline was associated with a lower risk of mortality (HR 0.95 [95% CI: 0.91, 0.98] per 1‐unit increase) (Table 2). The same variables were selected by all the algorithms.

### Sensitivity analyses assessing handling of missing data

In the INPULSIS trials, the variables selected when the multiple imputation method was used to impute missing data were consistent with the original analysis. Relative decline in DLCO >15% predicted within 12 months was selected as an additional variable when the jump to reference and copy reference methods were used to impute missing data (see Table S3 in the Supporting Information). When the jump to reference method was used, age was only selected as a variable when the forward selection algorithm was used. In the INBUILD trial, the same variables were selected when all three imputation methods were used as in the original analysis (see Table S4 in the Supporting Information).

### Association between alternative categorical declines in FVC and mortality

In the INPULSIS trials, all three alternative measures of decline in FVC were consistently selected when they replaced relative decline in FVC >10% predicted within 12 months in the model (see Tables S5–S10 in the Supporting Information). In the INBUILD trial, none of the alternative measures of FVC decline was selected using the stepwise or backward selection algorithms when they replaced relative decline in FVC >10% predicted within 12 months in the model (see Tables S11–S14 in the Supporting Information). Decline in FVC >5% predicted within 12 months was selected when forward selection was used.

When all four decline in FVC variables were included in a model with all other variables, none of the alternative measures was selected in place of relative decline in FVC >10% predicted within 12 months in INPULSIS or INBUILD (see Tables S15 and S16 in the Supporting Information). When all four decline in FVC variables were included without any other variables, relative decline in FVC >10% predicted within 12 months was selected using all three selection algorithms in the INPULSIS trials (HR 4.54 [95% CI: 2.76, 7.46]; *p* < 0.0001) and the INBUILD trial (HR 2.91 [95% CI: 1.43, 5.90]; *p* = 0.0031) (see Tables S17 and S18 in the Supporting Information).

## DISCUSSION

We used data from the INPULSIS trials in subjects with IPF and the INBUILD trial in subjects with progressive fibrosing ILDs other than IPF to investigate the impact of demographic and clinical variables on mortality over 52 weeks. In both the INPULSIS and INBUILD trials, a relative decline in FVC of >10% predicted within 12 months had the strongest association with mortality: compared with subjects who did not have such a decline, this decline in FVC increased the risk of death over 52 weeks by 3.8‐fold in subjects with IPF and by 2.6‐fold in subjects with other progressive fibrosing ILDs. This is supportive of previous studies that have consistently demonstrated an increased risk of mortality in patients with fibrosing ILDs whose FVC % predicted declined from baseline by 10% within 12 months.^11^, ^14^, ^15^, ^22^, ^23^, ^24^

A relative decline in FVC >5% predicted within 6 or 12 months and a relative decline in FVC >10% predicted within 6 months were associated with mortality when they replaced a relative decline in FVC >10% predicted within 12 months in the model in our analyses of the INPULSIS trials, but not in the analyses of the INBUILD trial. This might have been due to the lower number of FVC declines and deaths observed in the INBUILD trial than in the INPULSIS trials. In the INPULSIS trials, the other categorical declines in FVC were not as strongly associated with mortality as a relative decline in FVC >10% predicted within 12 months. Together with evidence from previous studies, these data support the use of a relative decline in FVC >10% predicted within 12 months as a robust predictor of mortality in patients with fibrosing ILDs.

Consistent with previous studies,^13^, ^18^, ^20^, ^24^, ^25^, ^26^ in our analyses, lower DLCO % predicted at baseline was associated with a higher risk of mortality over 52 weeks. Interestingly, lower FVC % predicted at baseline, which has been associated with mortality in other studies in subjects with IPF^20^, ^25^, ^27^ and with other fibrosing ILDs,^3^, ^14^, ^16^, ^23^ was identified as a factor associated with mortality in the INPULSIS trials, but not in the INBUILD trial. This may reflect the inclusion criteria used in the INBUILD trial, which required recent progression of ILD based on decline in FVC or worsening of symptoms and abnormalities on HRCT within the 24 months prior to enrolment, and/or the difference in FVC % predicted at baseline, which was higher in the INPULSIS trials than in the INBUILD trial (mean [SD] 79.6 [17.8] vs. 69.0 [15.6] % predicted). In the INPULSIS and INBUILD trials, nintedanib reduced the rate of decline in FVC compared to placebo.^5^, ^6^ The confounding effect of the association between treatment with nintedanib and reduced FVC decline meant that treatment with nintedanib was not selected as a variable associated with reduced mortality in these analyses.

The presence of a UIP‐like fibrotic pattern on HRCT has been associated with higher mortality in subjects with fibrosing ILDs,^13^, ^17^, ^28^, ^29^, ^30^ although this has not been observed in all studies.^3^ In the INBUILD trial, subjects who had a UIP‐like fibrotic pattern on HRCT had approximately a three‐fold higher risk of mortality over 52 weeks than subjects with other fibrotic patterns. However, it should be noted that placebo‐treated subjects with other fibrotic patterns on HRCT still had a marked decline in FVC over 52 weeks (−154 ml) and that based on data from the whole INBUILD trial (median follow‐up: approximately 19 months), a numerical association was observed between a relative decline in FVC >10% predicted and mortality in these subjects (HR 2.88).^8^

In our analyses, ILD diagnosis (idiopathic non‐specific interstitial pneumonia, unclassifiable idiopathic interstitial pneumonia, hypersensitivity pneumonitis, autoimmune ILDs, other ILDs) was not associated with mortality. Previous analyses of data from the INBUILD trial suggest that the rate of decline in FVC was consistent across subgroups by ILD diagnosis.^8^ This suggests that once a fibrosing ILD has become progressive, the risk of mortality that it confers is similar irrespective of aetiology. It is important to bear in mind that these conclusions are based on a population of patients with fibrosing ILD that had shown progression within the prior 24 months and should not be applied to patients at diagnosis; an accurate initial differential diagnosis of ILD remains essential.

Strengths of our analyses include the large number of subjects; the large quantity and high quality of FVC measurements collected in the setting of clinical trials; and the consistency of the findings across model selection algorithms and sensitivity analyses that investigated the impact of missing data. Limitations of our analyses include that interactions between variables were not evaluated and the small number of deaths observed over 52 weeks. The mortality rate observed in the placebo group of the INPULSIS trials was lower than that observed in large observational studies in patients with IPF,^31^, ^32^, ^33^ presumably as patients with the most severe disease or certain comorbidities were not enrolled. Acute exacerbations could not be considered as a factor associated with mortality in these analyses due to the differing definitions used in the INPULSIS and INBUILD trials. The potential association between patient‐reported outcomes and mortality was not evaluated.

In conclusion, these analyses of data from the INPULSIS and INBUILD trials provide further support for similarity in the course of lung injury between patients with IPF and with other forms of progressive fibrosing ILD and for the value of FVC decline as a predictor of short‐term mortality in these patients.

## CONFLICT OF INTEREST

*Research funding*: The INPULSIS and INBUILD trials were funded by Boehringer Ingelheim International GmbH. The authors did not receive payment for development of this article. Writing support was provided by Julie Fleming and Wendy Morris of Fleishman‐Hillard, London, UK, which was contracted and funded by Boehringer Ingelheim. Boehringer Ingelheim was given the opportunity to review the article for medical and scientific accuracy as well as intellectual property considerations.

Kevin K. Brown reports grants from NHLBI; has served on a Data Monitoring Committee for Biogen and Humanetics; has served as an advisor to AbbVie, Blade Therapeutics, Boehringer Ingelheim, Bristol‐Myers Squibb, CSL Behring, DevPro Biopharma, Dispersol, Galapagos, Galecto, Huitai Biomedicine, Lilly, the Open Source Imaging Consortium, Pliant, Redx Pharma, Sanofi, Theravance, Third Pole and Translate Bio; and reports scientific collaboration with Sekisui Medical Co. Yoshikazu Inoue reports grants from the Japan Agency for Medical Research and Development and the Japanese Ministry of Health, Labour, and Welfare; and payment for lectures or advisory boards from Asahi Kasei, Boehringer Ingelheim, Galapagos, Roche, Savara Inc., Shionogi & Co, Ltd and Taiho. Kevin R. Flaherty reports grants from Boehringer Ingelheim and Roche/Genentech and consulting fees from Bellerophon, Blade Therapeutics, Boehringer Ingelheim, DevPro, Horizon Pharmaceuticals, PureHealth, Respivant, Roche/Genentech, Shionogi & Co, Ltd and United Therapeutics. Fernando J. Martinez has served on a Data Safety Monitoring Board, advisory board or steering committee for Afferent/Merck, Bayer, Biogen, Boehringer Ingelheim, Nitto, Respivant, Roche and Veracyte; and has received consulting fees or payment for presentations from AbbVie, Boehringer Ingelheim, Bristol‐Myers Squibb, Bridge Biotherapeutics, CSL Behring, DevPro, IQVIA, Roche/Genentech, Sanofi, Shionogi & Co, Ltd, twoXAR, United Therapeutics and Veracyte. Vincent Cottin reports grants from Boehringer Ingelheim; consulting or lecturing fees from Actelion, AstraZeneca, Boehringer Ingelheim, Novartis, Roche/Promedior and Sanofi; support for attending meetings from Actelion, Boehringer Ingelheim and Roche/Promedior; and has served on a steering committee, advisory board, Data Safety Monitoring Board or adjudication committee for Actelion, Bayer/Merck Sharp & Dohme, Bristol‐Myers Squibb, Celgene, FibroGen, Galapagos, Galecto, Novartis, Roche/Promedior and Shionogi and Co, Ltd. Francesco Bonella has received speakers fees from Boehringer Ingelheim, Fujirebio, Galapagos and Roche; support for attending meetings from Boehringer Ingelheim and Roche; and has acted as an advisor for Boehringer Ingelheim, Bristol‐Myers Squibb, Fujirebio, Galapagos, GlaxoSmithKline, Roche and Takeda. Stefania Cerri reports payment for presentations from Boehringer Ingelheim. Sonye K. Danoff has been a clinical trial investigator or has served on a clinical trial coordinating committee for Boehringer Ingelheim, Bristol‐Myers Squibb and Roche/Genentech; has received consulting fees, presenting fees or support for attending meetings from Boehringer Ingelheim, the France Foundation and Lupin Pharma; has served on a Data Safety Monitoring Board or advisory board for Galecto and Galapagos; and has acted as an advisor for the American Thoracic Society and Pulmonary Fibrosis Foundation. Stephane Jouneau reports grants from AIRB, Boehringer Ingelheim, LVL, Novartis and Roche; payment for lectures or speaker bureaus from Actelion, AIRB, AstraZeneca, Boehringer Ingelheim, Bristol‐Myers Squibb, Chiesi, Genzyme, GlaxoSmithKline, LVL, MundiPharma, Novartis, Pfizer, Roche and Sanofi; support for attending meetings from Boehringer Ingelheim and Roche; and participation on advisory boards for Boehringer Ingelheim, Novartis and Roche. Rainer‐Georg Goeldner, Martin Schmidt and Susanne Stowasser are employees of Boehringer Ingelheim. Rozsa Schlenker‐Herceg was an employee of Boehringer Ingelheim at the time this research was conducted. Athol U. Wells reports consulting or speaker fees from Blade Therapeutics, Boehringer Ingelheim and Roche.

## AUTHOR CONTRIBUTION

**Kevin K. Brown:** Conceptualization (equal); visualization (supporting); writing – original draft (supporting); writing – review and editing (supporting). **Yoshikazu Inoue:** Conceptualization (supporting); investigation (equal); visualization (supporting); writing – review and editing (supporting). **Kevin R. Flaherty:** Conceptualization (supporting); visualization (supporting); writing – review and editing (supporting). **Fernando J. Martinez:** Conceptualization (supporting); visualization (supporting); writing – review and editing (supporting). **Vincent Cottin:** Conceptualization (supporting); investigation (equal); visualization (supporting); writing – review and editing (supporting). **Francesco Bonella:** Investigation (equal); visualization (supporting); writing – review and editing (supporting). **Stefania Cerri:** Investigation (equal); visualization (supporting); writing – review and editing (supporting). **Sonye K. Danoff:** Investigation (equal); visualization (supporting); writing – review and editing (supporting). **Stephane Jouneau:** Investigation (equal); visualization (supporting); writing – review and editing (supporting). **Rainer‐Georg Goeldner:** Conceptualization (equal); data curation (equal); formal analysis (lead); methodology (equal); visualization (equal); writing – original draft (supporting); writing – review and editing (supporting). **Martin Schmidt:** Data curation (equal); methodology (equal); visualization (supporting); writing – review and editing (supporting). **Susanne Stowasser:** Conceptualization (supporting); visualization (supporting); writing – review and editing (supporting). **Rozsa Schlenker‐Herceg:** Conceptualization (equal); project administration (lead); supervision (lead); visualization (equal); writing – original draft (lead); writing – review and editing (supporting). **Athol U. Wells:** Conceptualization (equal); visualization (supporting); writing – original draft (supporting); writing – review and editing (supporting).

## HUMAN ETHICS APPROVAL DECLARATION

The INBUILD and INPULSIS trials were conducted in accordance with the principles of the Declaration of Helsinki and the Harmonized Tripartite Guideline for Good Clinical Practice from the International Conference on Harmonization, and were approved by local authorities. The protocols were approved by an Independent Ethics Committee and/or Institutional Review Board at all the participating centres. All subjects provided written informed consent.

Clinical trial registration: INBUILD trial (NCT02999178) and INPULSIS trials (NCT01335464, NCT01335477) at clinicaltrials.gov.

## Supporting information


**Table S1.** Variables selected as being associated with mortality based on data from the INPULSIS trials.
**Table S2.** Variables selected as being associated with mortality based on data from the INBUILD trial.
**Table S3.** Selected variables and their association with mortality in the INPULSIS trials according to the imputation method.
**Table S4.** Selected variables and their association with mortality in the INBUILD trial according to the imputation method.
**Table S5.** Variables selected as being associated with mortality based on data from the INPULSIS trials when decline in FVC >10% predicted within 12 months was replaced by decline in FVC >10% predicted within 6 months in the model.
**Table S6.** Variables selected as being associated with mortality based on data from the INPULSIS trials when decline in FVC >10% predicted within 12 months was replaced by decline in FVC >5% predicted within 12 months in the model.
**Table S7.** Variables selected as being associated with mortality based on data from the INPULSIS trials when decline in FVC >10% predicted within 12 months was replaced by decline in FVC >5% predicted within 6 months in the model.
**Table S8.** Association between selected variables and mortality based on data from the INPULSIS trials when decline in FVC >10% predicted within 12 months was replaced by decline in FVC >10% predicted within 6 months in the model.
**Table S9.** Association between selected variables and mortality based on data from the INPULSIS trials when decline in FVC >10% predicted within 12 months was replaced by decline in FVC >5% predicted within 12 months in the model.
**Table S10.** Association between selected variables and mortality based on data from the INPULSIS trials when decline in FVC >10% predicted within 12 months was replaced by decline in FVC >5% predicted within 6 months in the model.
**Table S11.** Variables selected as being associated with mortality based on data from the INBUILD trial when decline in FVC >10% predicted within 12 months was replaced by decline in FVC >10% predicted within 6 months in the model.
**Table S12.** Variables selected as being associated with mortality based on data from the INBUILD trial when decline in FVC >10% predicted within 12 months was replaced by decline in FVC >5% predicted within 12 months in the model.
**Table S13.** Variables selected as being associated with mortality based on data from the INBUILD trial when decline in FVC >10% predicted within 12 months was replaced by decline in FVC >5% predicted within 6 months in the model.
**Table S14.** Association between selected variables and mortality based on data from the INBUILD trial when decline in FVC >10% predicted within 12 months was replaced by decline in FVC >10% predicted within 6 months, decline in FVC >5% predicted within 12 months or decline in FVC >5% predicted within 6 months in the model.
**Table S15.** Variables selected as being associated with mortality based on data from the INPULSIS trials when decline in FVC >10% predicted within 12 months, decline in FVC >10% predicted within 6 months, decline in FVC >5% predicted within 12 months and decline in FVC >5% predicted within 6 months were included in a model with all other variables.
**Table S16.** Variables selected as being associated with mortality based on data from the INBUILD trial when decline in FVC >10% predicted within 12 months, decline in FVC >10% predicted within 6 months, decline in FVC >5% predicted within 12 months and decline in FVC >5% predicted within 6 months were included in a model with all other variables.
**Table S17.** Variables selected as being associated with mortality based on data from the INPULSIS trials when decline in FVC >10% predicted within 12 months, decline in FVC >10% predicted within 6 months, decline in FVC >5% predicted within 12 months and decline in FVC >5% predicted within 6 months were included in a model with no other variables.
**Table S18.** Variables selected as being associated with mortality based on data from the INBUILD trial when decline in FVC >10% predicted within 12 months, decline in FVC >10% predicted within 6 months, decline in FVC >5% predicted within 12 months and decline in FVC >5% predicted within 6 months were included in a model with no other variables.

## Data Availability

To ensure independent interpretation of clinical study results, Boehringer Ingelheim grants all external authors access to relevant material, including participant‐level clinical study data, as needed by them to fulfil their role and obligations as authors under the ICMJE criteria. Clinical study documents and participant clinical study data are available to be shared on request after publication of the primary manuscript in a peer‐reviewed journal, and if regulatory activities are complete and other criteria met as per the BI Policy on Transparency and Publication of Clinical Study Data (https://www.mystudywindow.com/msw/datasharing). Bona fide, qualified scientific and medical researchers are eligible to request access to the clinical study data with corresponding documentation describing the structure and content of the data sets. Upon approval, and governed by a legal agreement, data are shared in a secured data‐access system for a period of 1 year, which may be extended upon request. Prior to providing access, clinical study documents and data will be examined, and, if necessary, redacted and de‐identified, to protect the personal data of study participants and personnel, and to respect the boundaries of informed consent. Researchers should use the https://vivli.org/ link to request access to study data and visit https://www.mystudywindow.com/msw/datasharing.
