# Supplementary material for: Predictors of mortality in subjects with progressive fibrosing interstitial lung diseases
Source: Respirology. 2022 Feb 27;27(4):294–300. doi: 10.1111/resp.14231 (PMC9306931; doi:10.1111/resp.14231)
Supplement: Supplementary file 1 — Table S1. Variables selected as being associated with mortality based on data from the INPULSIS trials. Table S2. Variables selected as being associated with mortality based on data from the INBUILD trial. Table S3. Selected variables and their association with mortality in the INPULSIS trials according to the imputation method. Table S4. Selected variables and their association with mortality in the INBUILD trial according to the imputation method. Table S5. Variables selected as being associated with mortality based on data from the INPULSIS trials when decline in FVC >10% predicted within 12 months was replaced by decline in FVC >10% predicted within 6 months in the model. Table S6. Variables selected as being associated with mortality based on data from the INPULSIS trials when decline in FVC >10% predicted within 12 months was replaced by decline in FVC >5% predicted within 12 months in the model. Table S7. Variables selected as being associated with mortality based on data from the INPULSIS trials when decline in FVC >10% predicted within 12 months was replaced by decline in FVC >5% predicted within 6 months in the model. Table S8. Association between selected variables and mortality based on data from the INPULSIS trials when decline in FVC >10% predicted within 12 months was replaced by decline in FVC >10% predicted within 6 months in the model. Table S9. Association between selected variables and mortality based on data from the INPULSIS trials when decline in FVC >10% predicted within 12 months was replaced by decline in FVC >5% predicted within 12 months in the model. Table S10. Association between selected variables and mortality based on data from the INPULSIS trials when decline in FVC >10% predicted within 12 months was replaced by decline in FVC >5% predicted within 6 months in the model. Table S11. Variables selected as being associated with mortality based on data from the INBUILD trial when decline in FVC >10% predicted within 12 months was replac [file RESP-27-294-s001.docx]

**SUPPORTING INFORMATION**

**Predictors of mortality in subjects with progressive fibrosing interstitial lung diseases**

Kevin K Brown, MD;^1^ Yoshikazu Inoue, MD;^2^ Kevin R Flaherty, MD;^3^ Fernando J Martinez, MD;^4^ Vincent Cottin, MD;^5^ Francesco Bonella, MD;^6^ Stefania Cerri, MD;^7^ Sonye K Danoff, PhD;^8^ Stephane Jouneau, MD;^9^ Rainer-Georg Goeldner, PhD;^10^ Martin Schmidt, Dip Stats;^11^ Susanne Stowasser, MD;^12^ Rozsa Schlenker-Herceg, MD;^13^ Athol U Wells, MD^14^

^1^Department of Medicine, National Jewish Health, Denver, Colorado, USA;

^2^Clinical Research Center, National Hospital Organization Kinki-Chuo Chest Medical Center, Sakai City, Osaka, Japan;

^3^Division of Pulmonary and Critical Care Medicine, University of Michigan, Ann Arbor, Michigan, USA;

^4^Weill Cornell Medicine, New York, New York, USA;

^5^National Reference Centre for Rare Pulmonary Diseases, Louis Pradel Hospital, Hospices Civils de Lyon, Claude Bernard University Lyon 1, UMR 754, ERN-LUNG, Lyon, France;

^6^Center for interstitial and rare lung diseases, Department of Pneumology, Ruhrlandklinik University Hospital, University of Duisburg-Essen, Essen, Germany; ^7^Center for Rare Lung Disease - Azienda Ospedaliero-Universitaria Policlinico di Modena, Modena, Italy;

^8^Johns Hopkins Medicine, Baltimore, Maryland, USA;

^9^Department of Respiratory Medicine, Competences Centre for Rare Pulmonary Diseases, CHU Rennes, IRSET UMR 1085, univ Rennes, Rennes, France; ^10^Boehringer Ingelheim Pharma GmbH & Co. KG, Biberach, Germany; ^11^Boehringer Ingelheim Pharma GmbH, Ingelheim am Rhein, Germany; ^12^Boehringer Ingelheim International GmbH, Ingelheim am Rhein, Germany; ^13^Boehringer Ingelheim Pharmaceuticals, Inc., Ridgefield, Connecticut, USA; ^14^National Institute for Health Research Respiratory Biomedical Research Unit, Royal Brompton and Harefield NHS Foundation Trust, and National Heart and Lung Institute, Imperial College, London, UK.

**Table S1.** Variables selected as being associated with mortality based on data from the INPULSIS trials

| **Selection algorithm** | **Step** | **Effect entered** | **Effect removed** | **Order of selection** | ***P*-value** |
| --- | --- | --- | --- | --- | --- |
| Stepwise selection | 1 | Relative decline in FVC >10% predicted within 12 months |  | 1 | <0.0001 |
|  | 2 | FVC % predicted at baseline |  | 2 | 0.0003 |
|  | 3 | Age |  | 3 | 0.0074 |
|  | 4 | DLco % predicted at baseline |  | 4 | 0.034 |
| Forward selection | 1 | Relative decline in FVC >10% predicted within 12 months |  | 1 | <0.0001 |
|  | 2 | FVC % predicted at baseline |  | 2 | 0.0003 |
|  | 3 | Age |  | 3 | 0.0074 |
|  | 4 | DLco % predicted at baseline |  | 4 | 0.034 |
| Backward selection | 1 |  | Sex | 12 | 0.99 |
|  | 2 |  | FVC mL at baseline | 11 | 0.78 |
|  | 3 |  | Relative decline in weight >5% within 12 months | 10 | 0.77 |
|  | 4 |  | Treatment (nintedanib/placebo) | 9 | 0.59 |
|  | 5 |  | Race | 8 | 0.53 |
|  | 6 |  | BMI (kg/m^2^) at baseline | 7 | 0.64 |
|  | 7 |  | Time since diagnosis (years) | 6 | 0.31 |
|  | 8 |  | Tobacco consumption | 5 | 0.29 |
|  | 9 |  | Relative decline in DLco >15% predicted within 12 months | 4 | 0.11 |

**Table S2.** Variables selected as being associated with mortality based on data from the INBUILD trial

| **Selection algorithm** | **Step** | **Effect entered** | **Effect removed** | **Order of selection** | ***P*-value** |
| --- | --- | --- | --- | --- | --- |
| Stepwise selection | 1 | Relative decline in FVC >10% predicted within 12 months |  | 1 | 0.0023 |
|  | 2 | DLco % predicted at baseline |  | 2 | 0.0063 |
|  | 3 | HRCT pattern |  | 3 | 0.011 |
| Forward selection | 1 | Relative decline in FVC >10% predicted within 12 months |  | 1 | 0.0023 |
|  | 2 | DLco % predicted at baseline |  | 2 | 0.0063 |
|  | 3 | HRCT pattern |  | 3 | 0.011 |
| Backward selection | 1 |  | Time since diagnosis (years) | 14 | 0.92 |
|  | 2 |  | FVC mL at baseline | 13 | 0.80 |
|  | 3 |  | Treatment (nintedanib/placebo) | 12 | 0.75 |
|  | 4 |  | Age | 11 | 0.61 |
|  | 5 |  | Tobacco consumption | 10 | 0.57 |
|  | 6 |  | FVC % predicted at baseline | 9 | 0.52 |
|  | 7 |  | BMI (kg/m^2^) at baseline | 8 | 0.45 |
|  | 8 |  | Relative decline in DLco >15% predicted within 12 months | 7 | 0.27 |
|  | 9 |  | Sex | 6 | 0.16 |
|  | 10 |  | ILD diagnosis | 5 | 0.16 |
|  | 11 |  | Relative decline in weight >5% within 12 months | 4 | 0.15 |
|  | 12 |  | Race | 3 | 0.13 |

**Table S3.** Selected variables and their association with mortality in the INPULSIS trials according to the imputation method

| **Imputation method** | **HR (95% CI)** | ***P*-value** |
| --- | --- | --- |
| No data imputation (original analysis) |  |  |
| Relative decline in FVC >10% predicted within 12 months | 3.77 (2.28, 6.24) | <0.0001 |
| Age | 1.03 (1.00, 1.07) | 0.037 |
| FVC % predicted at baseline | 0.97 (0.95, 0.99) | 0.0007 |
| DLco % predicted at baseline | 0.77 (0.61, 0.98) | 0.033 |
| Multiple imputation |  |  |
| Relative decline in FVC >10% predicted within 12 months | 4.26 (2.56, 7.09) | <0.0001 |
| Age | 1.03 (1.00, 1.07) | 0.042 |
| FVC % predicted at baseline | 0.97 (0.96, 0.99) | 0.0008 |
| DLco % predicted at baseline | 0.78 (0.62, 0.99) | 0.043 |
| Jump to reference |  |  |
| Relative decline in DLco >15% predicted within 12 months | 2.01 (1.19, 3.39) | 0.0088 |
| Relative decline in FVC >10% predicted within 12 months | 4.18 (2.48, 7.04) | <0.0001 |
| FVC % predicted at baseline | 0.98 (0.96, 0.99) | 0.0033 |
| DLco % predicted at baseline | 0.72 (0.57, 0.91) | 0.0057 |
| Copy reference |  |  |
| Relative decline in DLco >15% predicted within 12 months | 1.72 (1.02, 2.89) | 0.041 |
| Relative decline in FVC >10% predicted within 12 months | 4.27 (2.54, 7.18) | <0.0001 |
| Age | 1.03 (1.00, 1.07) | 0.0497 |
| FVC % predicted | 0.97 (0.96, 0.99) | 0.0010 |
| DLco % predicted | 0.76 (0.60, 0.97) | 0.024 |

In the multiple imputation and copy reference analyses, these variables were selected irrespective of whether the stepwise selection algorithm, forward selection algorithm or backwards selection algorithm was used. In the jump to reference analysis, age was only selected when using the forward selection algorithm (HR 1.03 [95% CI: 1.00, 1.07]; *P*=0.057).

**Table S4.** Selected variables and their association with mortality in the INBUILD trial according to imputation method

| **Imputation method** | **HR (95% CI)** | ***P*-value** |
| --- | --- | --- |
| No data imputation (original analysis) |  |  |
| HRCT pattern* | 2.98 (1.23, 7.22) | 0.016 |
| Relative decline in FVC >10% predicted within 12 months | 2.60 (1.28, 5.31) | 0.0085 |
| DLco % predicted at baseline | 0.95 (0.91, 0.98) | 0.0046 |
| Multiple imputation |  |  |
| HRCT pattern* | 2.99 (1.23, 7.25) | 0.015 |
| Relative decline in FVC >10% predicted within 12 months | 2.83 (1.38, 5.80) | 0.0045 |
| DLco % predicted at baseline | 0.95 (0.91, 0.98) | 0.0049 |
| Jump to reference |  |  |
| HRCT pattern* | 3.00 (1.24, 7.26) | 0.015 |
| Relative decline in FVC >10% predicted within 12 months | 3.23 (1.57, 6.68) | 0.0015 |
| DLco % predicted at baseline | 0.95 (0.92, 0.99) | 0.0061 |
| Copy reference |  |  |
| HRCT pattern* | 2.95 (1.22, 7.14) | 0.017 |
| Relative decline in FVC >10% predicted within 12 months | 3.16 (1.53, 6.53) | 0.0019 |
| DLco % predicted at baseline | 0.95 (0.92, 0.99) | 0.0060 |

These variables were selected irrespective of whether the stepwise selection algorithm, forward selection algorithm or backwards selection algorithm was used for each data imputation analysis. *HR for the UIP-like fibrotic pattern group compared with the other fibrotic patterns group.

**Table S5.** Variables selected as being associated with mortality based on data from the INPULSIS trials when decline in FVC >10% predicted within 12 months was replaced by decline in FVC >10% predicted within 6 months in the model

| **Selection algorithm** | **Step** | **Effect entered** | **Effect removed** | **Order of selection** | ***P*-value** |
| --- | --- | --- | --- | --- | --- |
| Stepwise selection | 1 | Relative decline in FVC >10% predicted within 6 months |  | 1 | <0.0001 |
|  | 2 | FVC % predicted at baseline |  | 2 | 0.0002 |
|  | 3 | DLco % predicted at baseline |  | 3 | 0.0053 |
|  | 4 | Age |  | 4 | 0.046 |
| Forward selection | 1 | Relative decline in FVC >10% predicted within 6 months |  | 1 | <0.0001 |
|  | 2 | FVC % predicted at baseline |  | 2 | 0.0002 |
|  | 3 | DLco % predicted at baseline |  | 3 | 0.0053 |
|  | 4 | Age |  | 4 | 0.046 |
| Backward selection | 1 |  | Sex | 12 | 0.97 |
|  | 2 |  | FVC mL at baseline | 11 | 0.75 |
|  | 3 |  | Relative decline in weight >5% within 12 months | 10 | 0.75 |
|  | 4 |  | Race | 9 | 0.52 |
|  | 5 |  | BMI (kg/m^2^) at baseline | 8 | 0.73 |
|  | 6 |  | Treatment (nintedanib/placebo) | 7 | 0.47 |
|  | 7 |  | Time since diagnosis (years) | 6 | 0.32 |
|  | 8 |  | Tobacco consumption | 5 | 0.28 |
|  | 9 |  | Relative decline in DLco >15% predicted within 12 months | 4 | 0.076 |

**Table S6.** Variables selected as being associated with mortality based on data from the INPULSIS trials when decline in FVC >10% predicted within 12 months was replaced by decline in FVC >5% predicted within 12 months in the model

| **Selection algorithm** | **Step** | **Effect entered** | **Effect removed** | **Order of selection** | ***P*-value** |
| --- | --- | --- | --- | --- | --- |
| Stepwise selection | 1 | FVC % predicted at baseline |  | 1 | <0.0001 |
|  | 2 | Relative decline in FVC >5% predicted within 12 months |  | 2 | 0.0011 |
|  | 3 | DLco % predicted at baseline |  | 3 | 0.0032 |
|  | 4 | Age |  | 4 | 0.027 |
|  | 5 | Relative decline in DLco >15% predicted within 12 months |  | 5 | 0.035 |
| Forward selection | 1 | FVC % predicted at baseline |  | 1 | <0.0001 |
|  | 2 | Relative decline in FVC >5% predicted within 12 months |  | 2 | 0.0011 |
|  | 3 | DLco % predicted at baseline |  | 3 | 0.0032 |
|  | 4 | Age |  | 4 | 0.027 |
|  | 5 | Relative decline in DLco >15% predicted within 12 months |  | 5 | 0.035 |
| Backward selection | 1 |  | FVC mL at baseline | 12 | 0.99 |
|  | 2 |  | Sex | 11 | 0.92 |
|  | 3 |  | Relative decline in weight >5% within 12 months | 10 | 0.73 |
|  | 4 |  | Race | 9 | 0.47 |
|  | 5 |  | BMI (kg/m^2^) at baseline | 8 | 0.60 |
|  | 6 |  | Treatment (nintedanib/placebo) | 7 | 0.42 |
|  | 7 |  | Tobacco consumption | 6 | 0.41 |
|  | 8 |  | Time since diagnosis (years) | 5 | 0.22 |

**Table S7.** Variables selected as being associated with mortality based on data from the INPULSIS trials when decline in FVC >10% predicted within 12 months was replaced by decline in FVC >5% predicted within 6 months in the model

| **Selection algorithm** | **Step** | **Effect entered** | **Effect removed** | **Order of selection** | ***P*-value** |
| --- | --- | --- | --- | --- | --- |
| Stepwise selection | 1 | FVC % predicted at baseline |  | 1 | <0.0001 |
|  | 2 | DLco % predicted at baseline |  | 2 | 0.0014 |
|  | 3 | Relative decline in FVC >5% predicted within 6 months |  | 3 | 0.0099 |
|  | 4 | Age |  | 4 | 0.030 |
|  | 5 | Relative decline in DLco >15% predicted within 12 months |  | 5 | 0.034 |
| Forward selection | 1 | FVC % predicted at baseline |  | 1 | <0.0001 |
|  | 2 | DLco % predicted at baseline |  | 2 | 0.0014 |
|  | 3 | Relative decline in FVC >5% predicted within 6 months |  | 3 | 0.0099 |
|  | 4 | Age |  | 4 | 0.030 |
|  | 5 | Relative decline in DLco >15% predicted within 12 months |  | 5 | 0.034 |
| Backward selection | 1 |  | FVC mL at baseline | 12 | 0.98 |
|  | 2 |  | Sex | 11 | 0.88 |
|  | 3 |  | Relative decline in weight >5% within 12 months | 10 | 0.70 |
|  | 4 |  | Race | 9 | 0.49 |
|  | 5 |  | BMI (kg/m^2^) at baseline | 8 | 0.62 |
|  | 6 |  | Tobacco consumption | 7 | 0.41 |
|  | 7 |  | Treatment (nintedanib/placebo) | 6 | 0.38 |
|  | 8 |  | Time since diagnosis (years) | 5 | 0.20 |

**Table S8.** Association between selected variables and mortality based on data from the INPULSIS trials when decline in FVC >10% predicted within 12 months was replaced by decline in FVC >10% predicted within 6 months in the model

| **Selected variables** | **HR (95% CI)** | ***P*-value** |
| --- | --- | --- |
| Relative decline in FVC >10% predicted within 6 months | 3.03 (1.86, 4.94) | <.0001 |
| Age | 1.03 (1.00, 1.07) | 0.046 |
| FVC % predicted at baseline | 0.97 (0.95, 0.99) | 0.0004 |
| DLco % predicted at baseline | 0.76 (0.59, 0.96) | 0.023 |

**Table S9.** Association between selected variables and mortality based on data from the INPULSIS trials when decline in FVC >10% predicted within 12 months was replaced by decline in FVC >5% predicted within 12 months in the model

| **Selected variables** | **HR (95% CI)** | ***P*-value** |
| --- | --- | --- |
| Relative decline in DLco >15% predicted within 12 months | 1.84 (1.04, 3.25) | 0.037 |
| Relative decline in FVC >5% predicted within 12 months | 2.19 (1.27, 3.78) | 0.0050 |
| Age | 1.04 (1.00, 1.07) | 0.030 |
| FVC % predicted at baseline | 0.97 (0.95, 0.99) | 0.0004 |
| DLco % predicted at baseline | 0.74 (0.58, 0.95) | 0.016 |

**Table S10.** Association between selected variables and mortality based on data from the INPULSIS trials when decline in FVC >10% predicted within 12 months was replaced by decline in FVC >5% predicted within 6 months in the model

| **Selected variables** | **HR (95% CI)** | ***P*-value** |
| --- | --- | --- |
| Relative decline in DLco >15% predicted within 12 months | 1.85 (1.04, 3.28) | 0.036 |
| Relative decline in FVC >5% predicted within 6 months | 1.83 (1.10, 3.05) | 0.020 |
| Age | 1.04 (1.00, 1.07) | 0.033 |
| FVC % predicted at baseline | 0.97 (0.95, 0.99) | 0.0004 |
| DLco % predicted at baseline | 0.74 (0.58, 0.95) | 0.016 |

**Table S11.** Variables selected as being associated with mortality based on data from the INBUILD trial when decline in FVC >10% predicted within 12 months was replaced by decline in FVC >10% predicted within 6 months in the model

| **Selection algorithm** | **Step** | **Effect entered** | **Effect removed** | **Order of selection** | ***P*-value** |
| --- | --- | --- | --- | --- | --- |
| Stepwise selection | 1 | DLco % predicted at baseline |  | 1 | 0.0031 |
|  | 2 | HRCT pattern |  | 2 | 0.014 |
| Forward selection | 1 | DLco % predicted at baseline |  | 1 | 0.0031 |
|  | 2 | HRCT pattern |  | 2 | 0.014 |
| Backward selection | 1 |  | Time since diagnosis (years) | 14 | 0.88 |
|  | 2 |  | FVC mL at baseline | 13 | 0.76 |
|  | 3 |  | Age | 12 | 0.69 |
|  | 4 |  | Treatment (nintedanib/placebo) | 11 | 0.65 |
|  | 5 |  | FVC % predicted at baseline | 10 | 0.53 |
|  | 6 |  | Tobacco consumption | 9 | 0.55 |
|  | 7 |  | BMI (kg/m^2^) at baseline | 8 | 0.45 |
|  | 8 |  | Relative decline in FVC >10% predicted within 6 months | 7 | 0.25 |
|  | 9 |  | Sex | 6 | 0.26 |
|  | 10 |  | Relative decline in weight >5% within 12 months | 5 | 0.15 |
|  | 11 |  | ILD diagnosis | 4 | 0.13 |
|  | 12 |  | Race | 3 | 0.10 |
|  | 13 |  | Relative decline in DLco >15% predicted within 12 months | 2 | 0.066 |

**Table S12.** Variables selected as being associated with mortality based on data from the INBUILD trial when decline in FVC >10% predicted within 12 months was replaced by decline in FVC >5% predicted within 12 months in the model

| **Selection algorithm** | **Step** | **Effect entered** | **Effect removed** | **Order of selection** | ***P*-value** |
| --- | --- | --- | --- | --- | --- |
| Stepwise selection | 1 | DLco % predicted at baseline |  | 1 | 0.0031 |
|  | 2 | HRCT pattern |  | 2 | 0.014 |
|  | 3 | Relative decline in FVC >5% predicted within 12 months |  | 3 | 0.049 |
|  | 4 |  | Relative decline in FVC >5% predicted within 12 months | 2 | 0.054 |
| Forward selection | 1 | DLco % predicted at baseline |  | 1 | 0.0031 |
|  | 2 | HRCT pattern |  | 2 | 0.014 |
|  | 3 | Relative decline in FVC >5% predicted within 12 months |  | 3 | 0.049 |
| Backward selection | 1 |  | Time since diagnosis (years) | 14 | 0.86 |
|  | 2 |  | FVC mL at baseline | 13 | 0.76 |
|  | 3 |  | Treatment (nintedanib/placebo) | 12 | 0.74 |
|  | 4 |  | Age | 11 | 0.72 |
|  | 5 |  | FVC % predicted at baseline | 10 | 0.58 |
|  | 6 |  | Tobacco consumption | 9 | 0.54 |
|  | 7 |  | BMI (kg/m^2^) at baseline | 8 | 0.46 |
|  | 8 |  | Sex | 7 | 0.25 |
|  | 9 |  | Relative decline in FVC >5% predicted within 12 months | 6 | 0.19 |
|  | 10 |  | Relative decline in weight >5% within 12 months | 5 | 0.15 |
|  | 11 |  | ILD diagnosis | 4 | 0.13 |
|  | 12 |  | Race | 3 | 0.10 |
|  | 13 |  | Relative decline in DLco >15% predicted within 12 months | 2 | 0.066 |

**Table S13.** Variables selected as being associated with mortality based on data from the INBUILD trial when decline in FVC >10% predicted within 12 months was replaced by decline in FVC >5% predicted within 6 months in the model

| **Selection algorithm** | **Step** | **Effect entered** | **Effect removed** | **Order of selection** | ***P*-value** |
| --- | --- | --- | --- | --- | --- |
| Stepwise selection | 1 | DLco % predicted at baseline |  | 1 | 0.0031 |
|  | 2 | HRCT pattern |  | 2 | 0.014 |
| Forward selection | 1 | DLco % predicted at baseline |  | 1 | 0.0031 |
|  | 2 | HRCT pattern |  | 2 | 0.014 |
| Backward selection | 1 |  | Time since diagnosis (years) | 14 | 0.83 |
|  | 2 |  | FVC mL at baseline | 13 | 0.75 |
|  | 3 |  | Age | 12 | 0.74 |
|  | 4 |  | Treatment (nintedanib/placebo) | 11 | 0.71 |
|  | 5 |  | FVC % predicted at baseline | 10 | 0.59 |
|  | 6 |  | Tobacco consumption | 9 | 0.54 |
|  | 7 |  | BMI (kg/m^2^) at baseline | 8 | 0.46 |
|  | 8 |  | Relative decline in FVC >5% predicted within 6 months | 7 | 0.36 |
|  | 9 |  | Sex | 6 | 0.26 |
|  | 10 |  | Relative decline in weight >5% within 12 months | 5 | 0.15 |
|  | 11 |  | ILD diagnosis | 4 | 0.13 |
|  | 12 |  | Race | 3 | 0.10 |
|  | 13 |  | Relative decline in DLco >15% predicted within 12 months | 2 | 0.066 |

**Table S14.** Association between selected variables and mortality based on data from the INBUILD trial when decline in FVC >10% predicted within 12 months was replaced by decline in FVC >10% predicted within 6 months, decline in FVC >5% predicted within 12 months or decline in FVC >5% predicted within 6 months in the model

| **Selected variables** | **HR (95% CI)** | ***P*-value** |
| --- | --- | --- |
| HRCT pattern* | 2.90 (1.20, 7.02) | 0.018 |
| DLco % predicted at baseline | 0.94 (0.91, 0.98) | 0.0022 |

These variables were selected when the stepwise selection and backward selection algorithm were used. Relative decline in FVC >5% predicted within 12 months was only selected when using the forward selection algorithm (HR 2.18 [95% CI: 0.99, 4.83]; *P*=0.054). *HR for the UIP-like fibrotic pattern group compared with the other fibrotic patterns group.

**Table S15.** Variables selected as being associated with mortality based on data from the INPULSIS trials when decline in FVC >10% predicted within 12 months, decline in FVC >10% predicted within 6 months, decline in FVC >5% predicted within 12 months and decline in FVC >5% predicted within 6 months were included in a model with all other variables

| **Selection algorithm** | **Step** | **Effect entered** | **Effect removed** | **Order of selection** | ***P*-value** |
| --- | --- | --- | --- | --- | --- |
| Stepwise selection | 1 | Relative decline in FVC >10% predicted within 12 months |  | 1 | <0.0001 |
|  | 2 | FVC % predicted at baseline |  | 2 | 0.0003 |
|  | 3 | Age |  | 3 | 0.0074 |
|  | 4 | DLco % predicted at baseline |  | 4 | 0.034 |
| Forward selection | 1 | Relative decline in FVC >10% predicted within 12 months |  | 1 | <0.0001 |
|  | 2 | FVC % predicted at baseline |  | 2 | 0.0003 |
|  | 3 | Age |  | 3 | 0.0074 |
|  | 4 | DLco % predicted at baseline |  | 4 | 0.034 |
| Backward selection | 1 |  | Sex | 15 | 0.97 |
|  | 2 |  | Relative decline in FVC >10% predicted within 6 months | 14 | 0.88 |
|  | 3 |  | FVC mL at baseline | 13 | 0.79 |
|  | 4 |  | Relative decline in weight >5% within 12 months | 12 | 0.80 |
|  | 5 |  | Treatment (nintedanib/placebo) | 11 | 0.56 |
|  | 6 |  | Race | 10 | 0.51 |
|  | 7 |  | BMI (kg/m^2^) at baseline | 9 | 0.64 |
|  | 8 |  | Relative decline in FVC >5% predicted within 12 months | 8 | 0.52 |
|  | 9 |  | Relative decline in FVC >5% predicted within 6 months | 7 | 0.58 |
|  | 10 |  | Time since diagnosis (years) | 6 | 0.31 |
|  | 11 |  | Tobacco consumption | 5 | 0.29 |
|  | 12 |  | Relative decline in DLco >15% predicted within 12 months | 4 | 0.11 |

**Table S16.** Variables selected as being associated with mortality based on data from the INBUILD trial when decline in FVC >10% predicted within 12 months, decline in FVC >10% predicted within 6 months, decline in FVC >5% predicted within 12 months and decline in FVC >5% predicted within 6 months were included in a model with all other variables

| **Selection algorithm** | **Step** | **Effect entered** | **Effect removed** | **Order of selection** | ***P*-value** |
| --- | --- | --- | --- | --- | --- |
| Stepwise selection | 1 | Relative decline in FVC >10% predicted within 12 months |  | 1 | 0.0023 |
|  | 2 | DLco % predicted at baseline |  | 2 | 0.0063 |
|  | 3 | HRCT pattern |  | 3 | 0.011 |
| Forward selection | 1 | Relative decline in FVC >10% predicted within 12 months |  | 1 | 0.0023 |
|  | 2 | DLco % predicted at baseline |  | 2 | 0.0063 |
|  | 3 | HRCT pattern |  | 3 | 0.011 |
| Backward selection | 1 |  | Time since diagnosis (years) | 17 | 0.88 |
|  | 2 |  | Treatment (nintedanib/placebo) | 16 | 0.81 |
|  | 3 |  | FVC mL at baseline | 15 | 0.83 |
|  | 4 |  | Tobacco consumption | 14 | 0.61 |
|  | 5 |  | Age | 13 | 0.56 |
|  | 6 |  | Relative decline in FVC >5% predicted within 6 months | 12 | 0.59 |
|  | 7 |  | Relative decline in FVC >5% predicted within 12 months | 11 | 0.73 |
|  | 8 |  | FVC % predicted at baseline | 10 | 0.57 |
|  | 9 |  | BMI (kg/m^2^) at baseline | 9 | 0.46 |
|  | 10 |  | Relative decline in FVC >10% predicted within 6 months | 8 | 0.26 |
|  | 11 |  | Relative decline in DLco >15% predicted within 12 months | 7 | 0.27 |
|  | 12 |  | Sex | 6 | 0.16 |
|  | 13 |  | ILD diagnosis | 5 | 0.16 |
|  | 14 |  | Relative decline in weight >5% within 12 months | 4 | 0.15 |
|  | 15 |  | Race | 3 | 0.13 |

**Table S17.** Variables selected as being associated with mortality based on data from the INPULSIS trials when decline in FVC >10% predicted within 12 months, decline in FVC >10% predicted within 6 months, decline in FVC >5% predicted within 12 months and decline in FVC >5% predicted within 6 months were included in a model with no other variables

| **Selection algorithm** | **Step** | **Effect entered** | **Effect removed** | **Order of selection** | ***P*-value** |
| --- | --- | --- | --- | --- | --- |
| Stepwise selection | 1 | Relative decline in FVC >10% predicted within 12 months |  | 1 | <0.0001 |
| Forward selection | 1 | Relative decline in FVC >10% predicted within 12 months |  | 1 | <0.0001 |
| Backward selection | 1 |  | Relative decline in FVC >10% predicted within 6 months | 3 | 0.82 |
|  | 2 |  | Relative decline in FVC >5% predicted within 12 months | 2 | 0.63 |
|  | 3 |  | Relative decline in FVC >5% predicted within 6 months | 1 | 0.69 |

**Table S18.** Variables selected as being associated with mortality based on data from the INBUILD trial when decline in FVC >10% predicted within 12 months, decline in FVC >10% predicted within 6 months, decline in FVC >5% predicted within 12 months and decline in FVC >5% predicted within 6 months were included in a model with no other variables

| **Selection algorithm** | **Step** | **Effect entered** | **Effect removed** | **Order of selection** | ***P*-value** |
| --- | --- | --- | --- | --- | --- |
| Stepwise selection | 1 | Relative decline in FVC >10% predicted within 12 months |  | 1 | 0.0021 |
| Forward selection | 1 | Relative decline in FVC >10% predicted within 12 months |  | 1 | 0.0021 |
| Backward selection | 1 |  | Relative decline in FVC >5% predicted within 6 months | 3 | 0.77 |
|  | 2 |  | Relative decline in FVC >5% predicted within 12 months | 2 | 0.49 |
|  | 3 |  | Relative decline in FVC >10% predicted within 6 months | 1 | 0.24 |
